# National Veterinary Medical Association

**Published:** 1886-10

**Authors:** 


### NATIONAL VETERINARY MEDICAL ASSOCIATION.

The 4th annual meeting of this association met in Columbus, Ohio, on September 2d. Dr. James Hamill, of New York, President, in the chair. The Ohio State Veterinary Association attended in a body. After attending to routine business and receiving reports, Dr. Fair of Cleveland, addressed the meeting on the protection and regulation of Veterinary Medicine, by state enactments and the modes of advertising by some members of the profession.

On opening the discussion on surgical cases, some members acknowledged their inability to perform certain operations, finding fault with the colleges for not giving sufficient clinical instruction. Dr. Hamill did not agree with this view in regard to one of the operations mentioned claiming that the colleges could not procure enough material of this nature for clinics, and that no member of the profession should be ashamed that he could not perform every operation on a horse, reminding the members that the medical profession were divided into various specialties and that some times the best physician would not perform a surgical operation.

The method of teaching at the various colleges was then explained by their respective graduates, which led to a lengthy debate as to what designation or title a veterinary surgeon should receive. Dr. Hamill stated that the title or degree given by the college was the proper one. The corporate body of Great Britain, which examined for the final degree of veterinary surgeon, gave the diploma of M. R. C. V. S., but the schools had only the power to grant the degree of veterinary surgeon. The New York College of Veterinary Surgeons, was founded on the same principle as the British schools, and its charter gives it the power to grant the degree of V. S.; when the American college was organized its trustees gave the graduates the title D. V. S., Doctor of Veterinary Surgery, and when the Columbia College was started the trustees gave the same D. V. S., by reading Doctor of Veterinary Science. The election of officers for the ensuing year resulted as follows : T. Bent Cotton, V.S., Mt. Vernon, Ohio, President; Prof. R. J. Withers, Chicago Vet. College; A. J. Chandler, V. S., Michigan; T. L. Armstrong, V. S., Indiana, Vice Presidents ; C. B. Robinson, V. S., Rec. Secretary ; James Hamill, New York, Cor. Secretary; L. V. Plageman, M. R. C. V. S., Brooklyn; New York, Treasurer. The evening session opened by Dr. W. F. Derr, of Wooster, Ohio, reading a paper on azoturia.

---

